# MIS technique for separation surgery in lumbar spine metastatic disease

**DOI:** 10.3171/2024.1.FOCVID23222

**Published:** 2024-04-01

**Authors:** Vicente de Paulo Martins Coelho Junior, Joravar S. Dhaliwal, Vikram B. Chakravarthy

**Affiliations:** Department of Neurological Surgery, The Ohio State University Wexner Medical Center, Columbus, Ohio

**Keywords:** minimally invasive spine surgery, preoperative embolization, separation surgery, spine metastasis, stereotactic radiotherapy

## Abstract

Around 40% of cancer patients present with spinal metastases (SM), the lumbar spine being the second most involved site (15%–30%) after the thoracic (60%–80%). Since the development of separation surgery, minimally invasive surgery (MIS) has increasingly been applied to approach SM, mirroring benefits yielded in the degenerative realm. Moreover, preoperative embolization potentially enhances local control for certain radioresistant histologies. Carbon fiber–reinforced PEEK hardware reduces image artifact, facilitating more accurate follow-up and radiotherapeutic planning. Additionally, short-segment cement-augmented constructs may be beneficial to decrease surgical morbidity and operative risk in this population. The authors present a lumbar spinal metastasis treated with MIS techniques.

The video can be found here: https://stream.cadmore.media/r10.3171/2024.1.FOCVID23222

## Transcript

In this video, we present a case of a lumbar spine metastatic lesion treated with a minimally invasive tubular resection and percutaneous pedicle screw fixation with cement augmentation and instrumented fusion.

### 0:33 Case Presentation.

A 59-year-old male presented with a 2-month history of progressive mechanical back pain with a left L2 radiculopathy compromising his gait and impacting his quality of life, reporting 10 out of 10 in the Numeric Rating Scale (NRS) for pain intensity.

On physical exam, he was intact, except for signs of a left L2 radiculopathy with a pain-limited left hip flexor weakness. Past medical record was positive for depression and arthritis, with no previous oncological history.

### 1:01 Imaging.

On the lumbar spine CT, a lytic lesion at the posterior left quadrant of the L2 vertebral body was noted, with a secondary compression fracture, with disruption of both endplates and with no burst component, along with the involvement of the left pedicle.

On MRI, we show parasagittal FLAIR sequences demonstrating bone marrow abnormalities at L2, with an axial T1 postcontrast sequence demonstrating the extraosseous extension of the tumor toward the epidural space, mainly the left lateral recess and foramen, and also involvement of the ipsilateral psoas muscle.

T2-weighted images showed the compression fracture at L2 and foraminal obliteration with compression of the exiting left L2 nerve root, correlating to patient’s presenting symptomatology.

Osseous lesions were also evidenced at C3 and T2, but with no significant epidural spinal cord compression.

Completion of systemic staging workup noted a left renal mass and multiple liver lesions.

### 2:05 Options for Management and Rationale.

Using the Spinal Instability Neoplastic Score (SINS) to assess tumor-related instability, it fell into the indeterminate category, totaling 10 points.

Given the visceral and osseous lesions, a needle biopsy was performed and confirmed adenocarcinoma. Despite conservative measures, his back pain and L2 radiculopathy persisted, significantly disrupting his quality of life. Various therapeutic approaches were discussed, including stereotactic radiation (SBRT) alone and also hybrid therapy, consisting of separation surgery followed by SBRT, including an open separation surgery versus a minimally invasive approach with short-segment construct and cement augmentation.^1–4^

The rationale behind separation surgery is to target circumferential decompression of the thecal sac, enabling delivery of an ablative dose of radiation, minimizing injury to the neural elements and preventing underdosing to the epidural space.

After a multidisciplinary spine tumor board discussion, the recommendation was to follow a palliative purpose, with a plan encompassing a preoperative tumor embolization,^5^ followed by separation surgery and SBRT for the L2 metastatic lesion.

Potential risks (including anesthetic-related complications, infection, hardware failure or even no clinical improvement) and benefits (as resolution of pain, better feasibility for a more effective adjuvant stereotactic radiation and less approach-related morbidity) were presented and patient agreed to pursue the indicated treatment.

### 3:28 Preoperative Embolization.

Twenty-four hours before the surgical procedure, a spinal angiogram showed a left-sided tumor blush centered at L2, with predominant vascular supply from the left L2 radicular arteries. PVA particles and coils were then utilized for embolization of those vessels, showing a marked reduction of tumoral blush.^5^

### 3:46 Surgical Steps.

The next day, the patient was brought to the OR. Some highlights about surgical setup: after induction of a total intravenous anesthesia, the patient was positioned prone on the open-frame Jackson table, and motor and somatosensory evoked potentials baselines were established. After prepping and draping in the usual fashion, a pin was inserted into the left posterior superior iliac spine for the navigation reference array, and then O-arm was brought to the field for an intraoperative CT scan.

Three incisions, 2 on the right and 1 on the left, were planned using navigation according to the optimal trajectories for screw placement. Using a navigated Jamshidi needle, pedicles at L1 and L3 were cannulated and K-wires inserted percutaneously. Carbon fiber screws with extenders were then placed and cement-augmented under fluoroscopy. Of note, coils in the left L2 radicular artery could be visualized.

We then docked a 22-mm tube on the left L2 lamina in the standard fashion using successive dilation.

Under microscopy, we resected muscle remnants over the L2 lamina and then performed an L2 left-sided hemilaminectomy using the M8 drill bit, along with a left L2–3 facetectomy and foraminotomy, removing yellow ligament with Kerrison rongeurs, exposing the thecal sac and the left L2 nerve root.

We then completed bony resection to reach the left L2 pedicle, which was infiltrated with tumor.

We proceeded with a piecemeal resection through the shoulder and axilla of the L2 root. We underscore the importance of finding the right plane and work through that interface when detaching the tumor from the neural elements, as demonstrated. Working with Woodson and down-pushing curettes, tumor debulking was carried out until achieving a complete circumferential decompression of the nerve root and thecal sac. Ideally, each traction or dissection maneuver vector should be performed away from neural elements, directing outward. The posterior longitudinal ligament can be cut and pushed into the bone defect, giving extra room to accomplish adequate separation.

Hemostasis was performed using a combination of bipolar, Surgiflo, and hydrogen peroxide. Under superficial magnification, here we can see that in the lumbar spine it is feasible to use the tubular approach even with the screw extenders in place.

We then placed carbon fiber rods percutaneously and final-tightened in a lordotic alignment. Estimated blood loss (EBL) was 200 ml and length of surgery was 4 hours.

### 7:06 Postoperative Course.

AP and lateral x-rays were obtained on postoperative day 1, showing adequate hardware placement. Patient had a significant improvement in his axial back pain and complete resolution of L2 radiculopathy, and was discharged on postoperative day 5 after achieving physical therapy goals.

At the 2-week postoperative check, the patient was doing well and all the incisions were healed, so he was cleared for radiation. Pathology showed metastatic carcinoma.

Here we see the radiotherapy planning, demonstrating the coil embolisate and highlighting the minimal hardware-related artifacts.

### 7:43 Background and Considerations on MIS for Metastatic Disease.

Around 40% of cancer patients present with metastatic spine involvement, being the lumbar spine the second most involved site (15%–30%) after the thoracic region (60%–80%).^2,3^ MIS philosophy has been increasingly applied to treat spinal metastases, mirroring some benefits yielded in the degenerative realm.^1,6^

SBRT changed the paradigm in this setting, providing effective results regardless of tumor histology and volume.^6^ Recent studies^2,7^ illustrated the effectiveness of hybrid therapy, eliminating the role of vertebrectomies for metastatic disease.^6^

Beyond facilitating surgical hemostasis, notably for renal and thyroid cancers, preoperative embolization potentially enhances local tumor control.^5^ It is essential to ensure an adequate embolization when considering MIS for highly vascular tumors, such as in this case.

Carbon fiber–reinforced PEEK hardware enables more accurate radiation planning and local control monitoring due to fewer artifact on imaging and reduced scattering effect on radiation.^8–10^ Moreover, compared to titanium, it has a lower elastic modulus which ultimately leads to less stress at the bone-hardware interface. This may also be advantageous as poor bone quality is a prevalent issue among cancer patients. In this setting, cement augmentation is one of the methods that have been studied in this population aiming at optimizing hardware stability and allowing for shorter constructs, ultimately working in synergy with an MIS tactic. This technique has demonstrated a twofold increase in screw pullout strength,^8,9^ and a recent analysis^4^ of short-segment cement-augmented constructs showed just one failure among 44 cases with an average follow-up of 10.7 months.

The quest for strategies to minimize the burden of a chosen treatment without disregarding oncological principles and biomechanical considerations about the construct longevity is fundamental in the decision-making for cancer population, where reduced survival further limits the ability to tolerate additional morbidity.

MIS techniques in metastatic disease may be used for select cases, such as when mechanical radiculopathy domains the clinical picture; when the pedicle or facet involvement is unilateral, and when just percutaneous stabilization would be required.

This case features a successful application of MIS, showing a blueprint to efficaciously deliver a less morbid treatment and simultaneously achieve established oncological goals.

## Disclosures

The authors report no conflict of interest concerning the materials or methods used in this study or the findings specified in this publication.

## Author Contributions

Primary surgeon: Chakravarthy. Assistant surgeon: Martins Coelho Junior, Dhaliwal. Editing and drafting the video and abstract: Martins Coelho Junior, Chakravarthy. Critically revising the work: Martins Coelho Junior, Chakravarthy. Reviewed submitted version of the work: all authors. Approved the final version of the work on behalf of all authors: Martins Coelho Junior.

## Supplemental Information

### Patient Informed Consent

The necessary patient informed consent was obtained in this study.
